# Furin inhibitor protects against neuronal cell death induced by activated NMDA receptors

**DOI:** 10.1038/s41598-018-23567-0

**Published:** 2018-03-26

**Authors:** Mariko Yamada, Hideki Hayashi, Moe Yuuki, Nahoko Matsushima, Bo Yuan, Norio Takagi

**Affiliations:** Department of Applied Biochemistry, Tokyo University of Pharmacy and Life Sciences, 1432-1 Horinouchi, Hachioji Tokyo, 192-0392 Japan

## Abstract

The proprotein convertases (PCs) act as serine proteases and are known to convert diverse precursor proteins into their active forms. Among the PCs, furin has been considered to play a crucial role not only in embryogenesis, but also in the initiation and progression of certain pathologic conditions. However, the roles played by furin with respect to neuronal cell injuries remain to be determined. An excessive influx of Ca^2+^ through the *N*-methyl-d-aspartate (NMDA) receptor has been associated with diverse neurological and neurodegenerative disorders. The aim of this study was to achieve further insight into the pathophysiologic roles of furin in cultured cortical neurons. We demonstrated that furin inhibitors dose-dependently prevented neuronal injury induced by NMDA treatment. Neuronal injury induced by NMDA treatment was attenuated by the calpain inhibitor calpeptin. And the increase observed in the activity of calpain after NMDA treatment was significantly inhibited by these furin inhibitors. Furthermore, calpain-2 activity, which was evaluated by means of the immunoblotting assay, was increased by NMDA treatment. It was noteworthy that this increased activity was almost completely inhibited by a furin inhibitor. Our findings suggested that furin is involved in NMDA-induced neuronal injury by acting upstream of calpain.

## Introduction

In the central nervous system, the ionotropic glutamate receptors, which are ligand-gated ion channels, play an important role in excitatory neurotransmission. Among these receptors, the *N*-methyl-d-aspartate (NMDA) receptor is highly permeable to calcium and sodium ions^1^. Whereas the activity of the NMDA receptor is associated with physiological functions such as learning and memory, an excessive influx of Ca^2+^ through the NMDA receptor has been related to diverse neurological and neurodegenerative disorders, including stroke, epilepsy, Parkinson’s disease, Alzheimer’s disease, and amyotrophic lateral sclerosis^2^. Therefore, it is an important objective to develop therapeutic strategies to prevent NMDA receptor-mediated excitotoxicity and to determine their underlying mechanisms.

The proprotein convertases (PCs) act as calcium-dependent serine proteases and convert various precursor proteins into their active forms^3^. The PCs constitute a family of 9 secretory serine proteases, including PC1/3, PC2, furin (PCSK3), PC4, PC5, paired basic amino acid-cleaving enzyme 4 (PACE4), PC7, subtilisin kexin isozyme 1 (SKI-1), and proprotein convertase subtilisin kexin 9 (PCSK9)^3,4^. In the secretory pathway, they are known to mediate the tissue-specific endoproteolytic activation of functional proteins, such as hormones, neuropeptides, growth factors and their receptors, adhesion molecules, bacterial toxins, and viral glycoproteins, by cleaving the inactive precursor proteins^4^. Therefore, altered activity of PCs has been implicated in several diseases such as Alzheimer’s disease^5^, endocrinopathies^6^ and cancer^7,8^.

Among the PCs, furin is ubiquitously expressed and trafficked to the trans-Golgi network or recycled to the cell surface through the endosomal system^9^. Also, furin controls the function of several types of proteins by cleaving inactive protein precursors in the secretory pathway^10,11^. Accumulating evidence indicates that furin may also play a pivotal role not only in embryogenesis, but also in the initiation and progression of certain pathologic conditions, including coronary artery disease, neurologic disease, and cancer^9,12^. However, the pathophysiological roles of furin in neuronal cell injuries induced by over-activated NMDA receptors remain to be determined. The aim of this study was to obtain further insight into the pathophysiologic roles of furin. Using cultured cortical neurons, we examined the effects of furin inhibitors on NMDA receptor-mediated neurotoxicity, which is associated with excessive Ca^2+^ influx into the neuron and has been implicated in a variety of neurodegenerative diseases.

## Results

**Figure 1:**
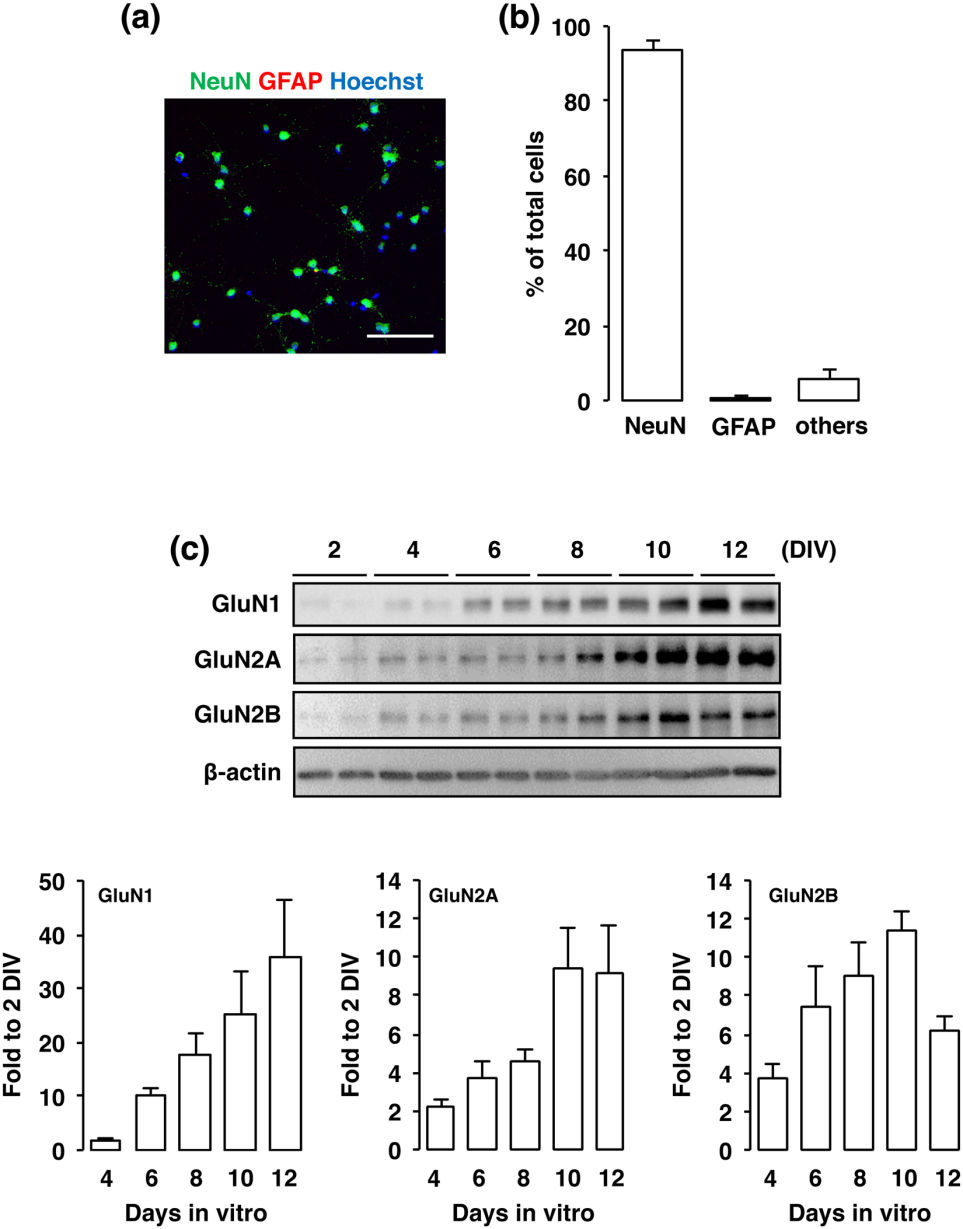
(**a**) Cortical cells were fixed at 10 DIV and stained for NeuN (green) and GFAP (red) and with Hoechst (blue). The scale bar represents 100 µm. (**b**) The number of NeuN- and GFAP-positive cells was counted. Results were expressed as the percentage of these cells among the total number of Hoechst-positive cells and as the means ± SE in 4 independent experiments. (**c**) Proteins from cultured cortical neurons at 2, 4, 6, 8, 10 and 12 DIV were analyzed by immunoblotting with anti-GluN1, anti-GluN2A, anti-GluN2B, and anti-β-actin antibodies. Bands corresponding to GluN1, GluN2A, and GluN2B were scanned, the scanned bands were normalized by each protein at 2 DIV on the same blot. β-actin was used as a loading control. Results are the means ± SE (n = 4 independent experiments).

**Figure 2:**
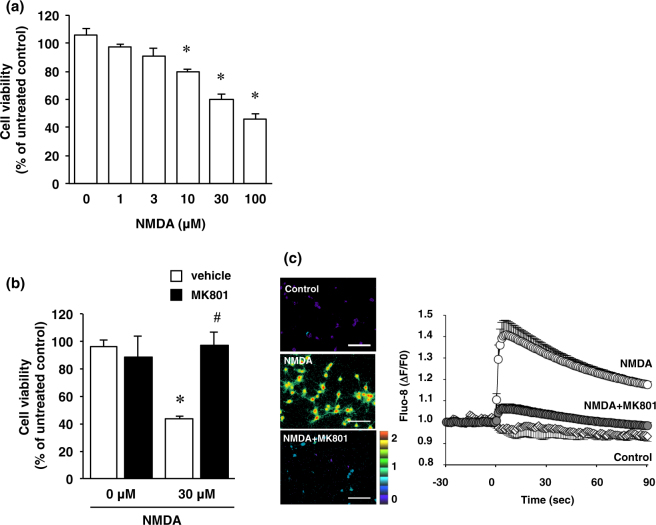
(**a**) Effect of NMDA treatment on cell viability. Cortical neurons were incubated with NMDA at the indicated concentrations for 15 minutes. After 24 h of NMDA treatment, cell viability was determined by performing the XTT dye-reduction assay. The absorbance at 450 nm was measured, and the relative cell viability was expressed as the percentage of the absorbance at 450 nm of each treatment group against that of the untreated control group. Results are the means ± SE (n = 5 independent experiments). *Indicates a significant difference from the NMDA-untreated group (*p* < 0.05). (**b**) Effect of NMDA receptor antagonist on NMDA-induced cell injury. Cell viability in cultures of 0 µM and 30 µM NMDA-treated cells without (white bars) or with (black bars) 10 µM MK-801. The relative cell viability was expressed as the percentage of the absorbance at 450 nm of each treatment group against that of the untreated control group. Results are the means ± SE (n = 3 independent experiments). *Indicates a significant difference from the NMDA-untreated group (*p* < 0.05); and ^#^, a significant difference from the NMDA-treated and MK801-untreated group (*p* < 0.05). (**c**) Cortical neurons were labeled with Fluo-8 acetoxymethyl ester for 30 min, and then 30 µM NMDA was added with or without 10 µM MK-801. Fluorescence ratio images are displayed in pseudocolor as indicated by the color bar. Pseudocolor represents changes in fluorescence ratios between 0 (*blue*) and 2 (*red*) corresponding to 1 (*green*), which is defined as the basal fluorescence intensity before NMDA stimulation. Representative ratio images of cortical neuron cultures incubated with 0 µM NMDA (Control), 30 µM NMDA (NMDA) or 30 µM NMDA and 10 µM MK-801 (NMDA + MK-801) are shown. Scale bar represents 100 µm. In the right panel, changes in Fluo-8 fluorescence are expressed as Δ*F*/*F*0, where *F*0 is basal fluorescence intensity before NMDA stimulation. Results are the means ± SE (n = 5 independent experiments).

In the present study, the primary cultures at 10 DIV contained 93.9 ± 2.1% NeuN-positive neurons and 0.6% ± 2.1% GFAP-positive astrocytes (Fig. 1a,b). NMDA receptors are heteromeric proteins that consist of combinations of GluN1 and GluN2, of which there are 4 types (GluN2A-D), or GluN3A subunits^13–17^. Whereas GluN1 is the principal subunit for NMDA receptor channel activity, GluN2 subunits determine the specificity of receptor function^18^. We next confirmed the presence of NMDA receptor subunits GluN1, GluN2A and GluN2B in cultured cortical neurons at 2, 4, 6, 8, 10, and 12 DIV. In agreement with the results of a previous study, NMDA receptor subunits were expressed in the cultured cortical neurons throughout the cultured period (Fig. 1c). Next, cortical neurons were exposed to various concentrations of NMDA (1–100 µM) for 15 min. In the XTT assay, the cell viability was decreased dose-dependently by such exposure (Fig. 2a). These decreases were significant at the concentrations of 10, 30 and 100 µM NMDA (Fig. 2a). The concentration of NMDA (30 µM) used in the following study was based on these data. We next certified that the decreased cell viability was caused by activation of the NMDA receptor by using the non-competitive NMDA receptor antagonist MK801 (Fig. 2b). Furthermore, we verified that the NMDA-induced increase in the intracellular Ca^2+^ concentration was mediated by the NMDA receptor (Fig. 2c).

**Figure 3:**
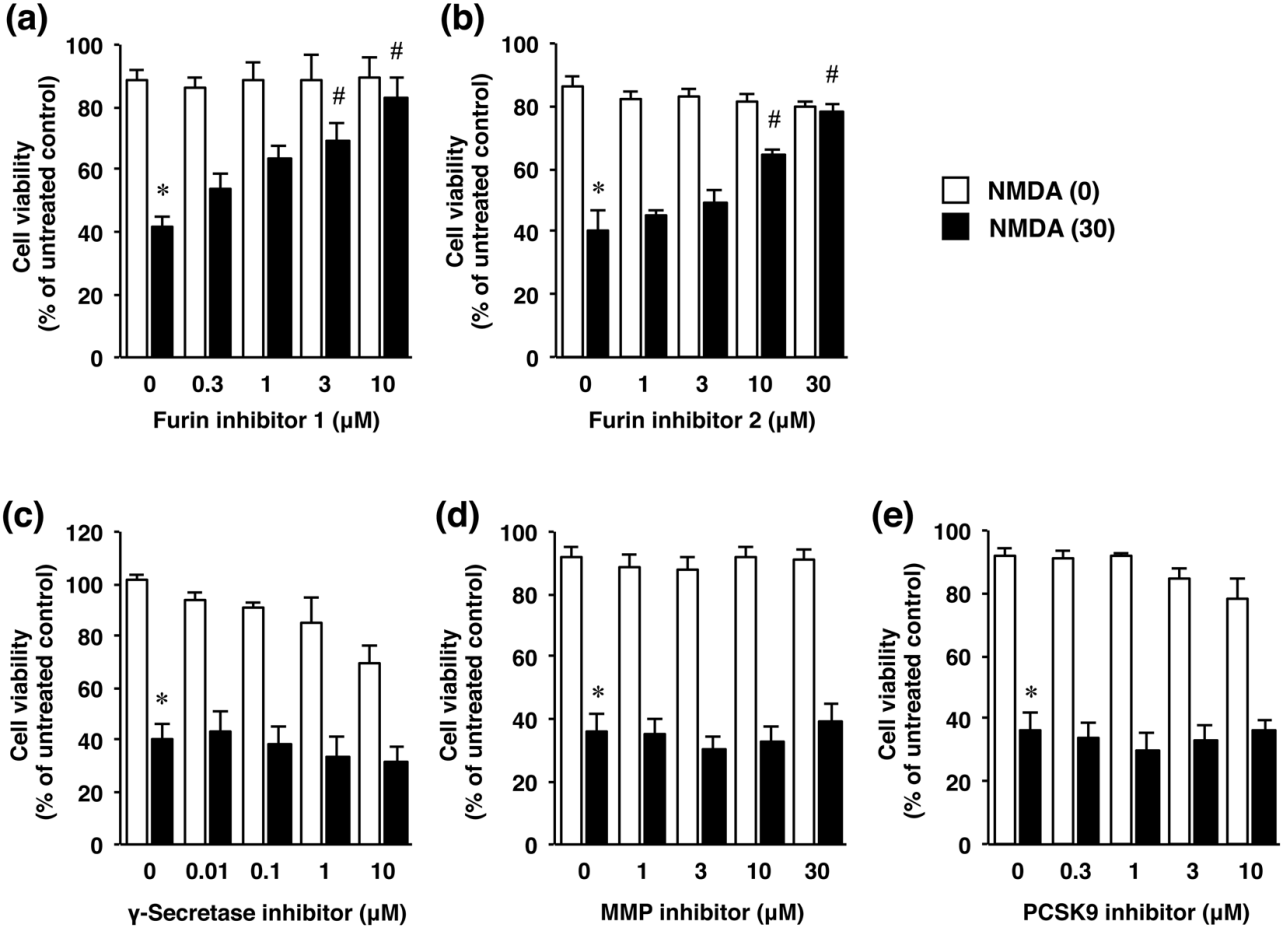
Effects of furin inhibitors (**a**,**b**) as well as several protease inhibitors (**c**–**e**) on NMDA-induced cell injury. Cell viability in cultures of cortical neurons treated with 0 µM (white bars) or 30 µM NMDA (black bars) without or with the indicated concentrations of furin inhibitor 1 (**a**), furin inhibitor 2 (**b**), γ-secretase inhibitor (**c**), MMP inhibitor (**d**) or PCSK9 inhibitor (**e**). The relative cell viability was expressed as the percentage of the absorbance at 450 nm of each treatment group against that of the untreated control group. Results are the means ± SE (n = 4 independent experiments). *Indicates a significant difference from the NMDA- and corresponding inhibitor-untreated group (*p* < 0.05); and ^#^, a significant difference from the NMDA-treated and furin inhibitor 1- untreated group (*p* < 0.05).

We next examined the effects of furin inhibitor 1 (Fig. 3a) and furin inhibitor 2 (Fig. 3b) as well as those of several protease inhibitors, including inhibitors for γ-secretase (Fig. 3c), matrix metalloproteinase (MMP; Fig. 3d), and PCSK9 (Fig. 3e) on NMDA-induced cortical cell injury. Treatment with either furin inhibitor dose-dependently prevented neuronal cell injury induced by the NMDA treatment; whereas inhibitors of γ-secretase, MMP, and PCSK9 were not preventive at the examined doses under this condition (Fig. 3).

**Figure 4:**
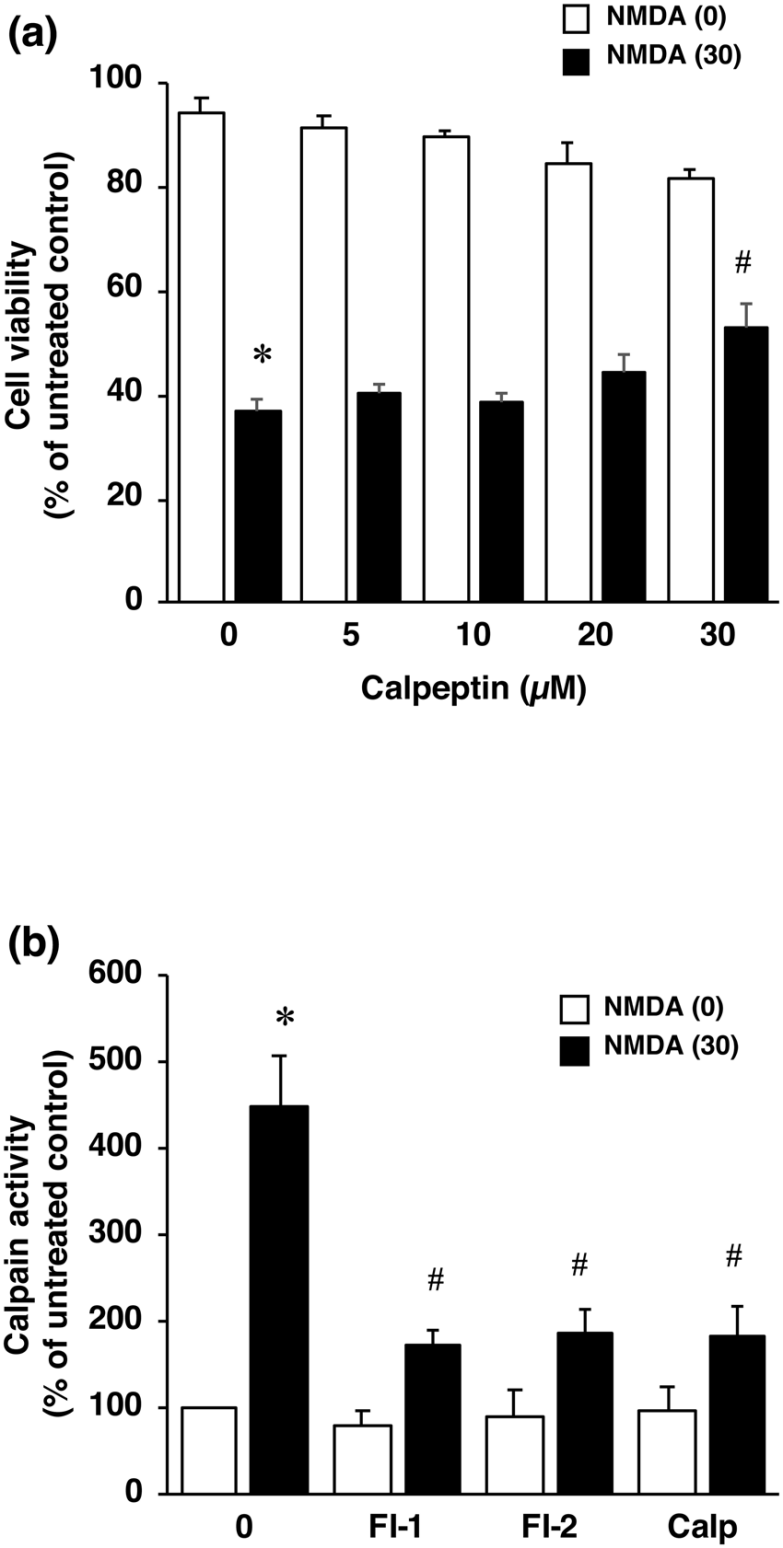
(**a**) Effect of calpeptin, a calpain inhibitor, on NMDA-induced cell injury. Cell viability in cortical neuron cultures treated with 0 µM (white bars) or 30 µM NMDA (black bars) without or with the indicated concentrations of calpeptin. The relative cell viability was expressed as the percentage of the absorbance at 450 nm of each treatment group against that of the untreated control group. Results are the means ± SE (n = 4 independent experiments). *Indicates a significant difference from the NMDA- and calpeptin-untreated group (*p* < 0.05); and ^#^, a significant difference from the NMDA-treated and calpeptin-untreated group (*p* < 0.05). (**b**) Effect of furin inhibitor 1 (FI-1) and 2 (FI-2) as well as that of calpeptin (Calp) on calpain activity after NMDA treatment. Calpain activity in cultures treated with 0 µM (white bars) or 30 µM (black bars) NMDA without or with 10 µM furin inhibitor 1, 30 µM furin inhibitor 2 or 30 µM calpeptin is shown. Calpain activity was expressed as the percentage of the untreated control group. Results are the means ± SE (n = 5 independent experiments). *Indicates a significant difference from the NMDA- and inhibitor-untreated group (*p* < 0.05); and ^#^, a significant difference from the NMDA-treated and inhibitor-untreated group (*p* < 0.05).

**Figure 5:**
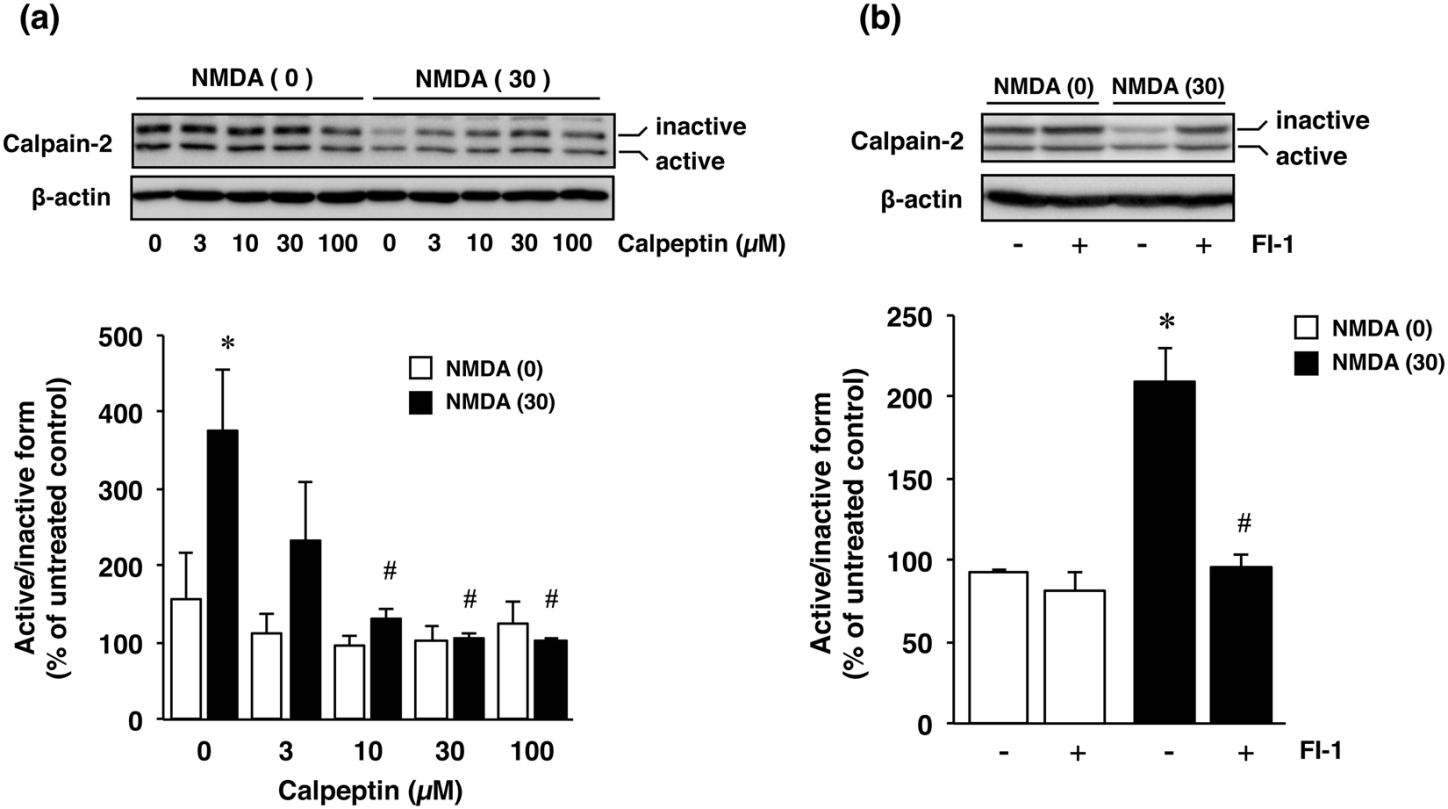
(**a**) Effect of calpeptin on NMDA-induced activation of calpain-2. Calpain-2 activity was evaluated according to the ratio of active-form/inactive-form band density determined by Western immunoblotting. Calpain-2 activity in cultures of 0 µM (white bars) or 30 µM NMDA- (black bars) treated cells without or with the indicated concentrations of calpeptin is indicated. Bands corresponding to calpain-2 (the ratio of active-form/inactive-form) and β-actin were scanned, and the scanned bands were normalized by β-actin on the same blot. Results are the means ± SE (n = 3 independent experiments). *Indicates a significant difference from the NMDA- and calpeptin-untreated group (*p* < 0.05); and ^#^, a significant difference from the NMDA-treated and calpeptin-untreated group (*p* < 0.05). (**b**) Effect of furin inhibitor on NMDA-induced activation of calpain-2. Calpain-2 activity was evaluated by Western immunoblotting as described above. Calpain-2 activity in cultures of 0 µM (white bars) and 30 µM NMDA- (black bars) treated cells without or with 10 µM furin inhibitor 1 (FI-1) is shown. Bands corresponding to calpain-2 (the ratio of active-form/inactive-form) and β-actin were scanned, and the scanned bands were normalized by β-actin on the same blot. Results are the means ± SE (n = 3 independent experiments). *Indicates a significant difference from the NMDA- and furin inhibitor 1 (FI-1)-untreated group (*p* < 0.05); and ^#^, a significant difference from the NMDA-treated and furin inhibitor 1 (FI-1)-untreated group (*p* < 0.05).

Next to determine the mechanism(s) of this protective effect of furin inhibitors against NMDA-induced neuronal cell injury, we focused on the calcium-activated protease calpain and at first assessed the effect of calpeptin, a calpain inhibitor, on cell viability. Treatment with calpeptin attenuated the cell injury induced by NMDA (Fig. 4a), thus indicating that calpain played a crucial role in NMDA-induced neuronal injury. Therefore, we next examined the effects of furin inhibitors on calpain activity by performing the Calpain-Glo assay. The results demonstrated that treatment with NMDA markedly activated calpain and that the increased activity was significantly inhibited by furin inhibitors as well as by calpeptin (Fig. 4b). Furthermore, calpain-2 activity was evaluated according to the ratio of active-form/inactive-form bands as determined by using the immunoblot assay (Fig. 5a). The calpain-2 activity was significantly increased by NMDA treatment, and this increased activation was inhibited by calpeptin in a concentration-dependent manner (Fig. 5a). We further examined whether furin inhibitor 1 would affect the increased activity of calpain-2 in NMDA-induced neurotoxicity. The results demonstrated that this furin inhibitor almost completely inhibited the increase in calpain-2 activity after treatment with NMDA (Fig. 5b).

## Discussion

It has been indicated that furin plays a pivotal role in embryogenesis and homeostasis. For example, the knockout of furin in a mouse model results in death at embryonic day 11, which is caused by hemodynamic insufficiency and defective cardiac ventral closure defects^19,20^. In addition, the expression of furin protein is increased in reactive astrocytes after oxygen-glucose deprivation (OGD) followed by reperfusion, likely resulting from up-regulation of hypoxia-inducible factor-1α^21^. This enhanced furin activity can contribute to the processing of proBDNF, leading to a maturation of BDNF (mBDNF) in reactive astrocytes; although other proprotein convertases may also have the ability to cleave proBDNF^21^. Consequently, an increased expression of mature BDNF by biological activation of furin may contribute to cell survival in response to a pathological condition such as OGD. In neurons, when proBDNF is not cleaved via appropriate proprotein processing, biological effects opposite those of mBDNF occur, such as neuronal apoptosis^22^. It was also reported that human immunodeficiency virus reduces furin levels mediated by gp120, leading to inhibition of the appropriate proBDNF processing into mature BDNF^23^. These findings imply that furin contributes to proBDNF processing, which might be associated with neuronal survival. In contrast, our findings demonstrated that treatment with furin inhibitors dose-dependently attenuated neuronal injury induced by NMDA treatment, whereas several other protease inhibitors, including inhibitors for γ-secretase, MMP and PCSK9 did not exert the protective effects, suggesting that activation of furin might contribute to NMDA receptor-associated pathologies. Although the role of furin under pathologic conditions remains controversial, accumulating evidence has revealed that furin can play a pivotal role in the initiation and progression of atherosclerosis through regulation of the inflammatory response, lipid metabolism, blood pressure, and formation of atheromatous plaques^24^. Therefore, furin has been proposed as a potential therapeutic target in various cancers and metastases, as this enzyme is often up-regulated in cancers and metastases^25^. In this sense, furin is also involved in the malignant phenotype of rhabdomyosarcoma cells by activating proteins^26^. Thus, these findings, including ours, suggest that furin has the ability to be a therapeutic target in some diseases. Whereas we demonstrated that the furin inhibitors prevented NMDA-induced neuronal cell injury as described above, the effect and its mechanisms have not been extensively studied. To further clarify the basis of the biological mechanism involved, we focused on a protease that plays a critical role under pathologic conditions by acting in a calcium-dependent manner. We showed that treatment with NMDA induced activation of calpain in cultured cortical neurons and that application of calpeptin, an inhibitor of calpain, attenuated NMDA-induced neuronal injuries. Consistent with previous studies^27^, calcium-dependent calpain activation was involved in NMDA-induced neuronal injury. Furin acts as a calcium-dependent proprotein convertase, raising the possibility that a pronounced increase in calcium influx through the NMDA receptor may activate furin, which may be associated with calpain-dependent neuronal injury under NMDA receptor-mediated neurotoxicity. It is noteworthy that NMDA-induced calpain activity was inhibited by treatment with furin inhibitors. Calpain-1 has been shown to localize in synaptic sites and to be preferentially activated by postsynaptic NMDA receptors^28^. In addition, calpain-1 activation at the synapse has been proposed to be required for induction of long-term potentiation and to contribute to the neuroprotective effects^28^. In contrast, extrasynaptic NMDA receptors have been shown to activate calpain-2, consequently leading to neurotoxicity^28,29^. Thus, we focused on the effects of furin inhibitors on the activity of calpain-2 under NMDA-induced toxicity. The results demonstrated that the increase in the activity of calpain-2 after treatment with NMDA was almost completely inhibited by furin inhibitor 1. These findings suggested that this furin inhibitor, at least in part, attenuated neuronal injury by inhibiting calpain-2 activity mediated by the extrasynaptic NMDA receptor. Calpain activation has been shown in both early and late phases of excitotoxicity in several neurodegenerative conditions^27,30^. In addition, it was recently reported that the activation of GluN2B-containing NMDA receptors followed by Ca^2+^ influx and activation of calpain resulted in rapid irreversible endocytosis of voltage-gated K^+^ channels Kv7.2 and Kv7.3 that likely represented an early response during excitotoxic states in hippocampal neuronal cultures^31^. Whereas, the activation of calpain might lead to later activation of the apoptotic proteases such as caspase-3 and caspase-7^32,33^. Therefore, future studies will be important to determine when furin regulates calpain activity, which ultimately leads to cell death. The NMDA receptor has been implicated in a variety of neurodegenerative disorders, including stroke, epilepsy, amyotrophic lateral sclerosis, and Alzheimer’s diseases. Therefore, it is important to determine the effect of furin inhibition and to explore the nature of intracellular signaling pathways under various NMDA-mediated pathophysiological conditions to develop appropriate therapeutic strategies for these diseases.

Calpain activity could be regulated by endogenous factors. Therefore, we cannot fully rule out the possibility that the furin inhibitors used in this study might have associated with other targets aside from furin to inhibit calpain activity or might have directly inhibited calpain activity under NMDA-induced neurotoxicity. In this sense, we sought to determine the direct effect of furin inhibitor on calpain activity *in vitro*. We observed that Ca^2+^-dependent activity of recombinant calpain was not inhibited by furin inhibitor, even if calpeptin significantly inhibited the activity of recombinant calpain in the presence of Ca^2+^ (supplementary Fig. S3). Therefore, it has a possibility that furin inhibitor has no direct effect on calpain activity under neurotoxicity, although future studies will be needed to determine the direct effect of other furin inhibitors on calpain activity of each isoform. Taken together, our findings are the first to demonstrate that furin is involved in NMDA-induced neuronal injury upstream of calpain. The results in the present study suggest that treatment with furin inhibitors could protect neurons against NMDA receptor-mediated excitotoxicity via the inhibition of calpain activity.

## Materials and Methods

### Materials

Furin inhibitor 1 (Cat. No. 14965) and SBC115076 (Cat. No. 19134) were purchased from Cayman Chemical (Ann Arbor, MI, USA). Furin inhibitor 2 (Cat. No. SCP0148) and MK-801 (Cat. No. M107) were obtained from Sigma Aldrich (St. Louis, MO, USA). GM6001 (Cat. No. 364206) and Calpeptin (Cat. No. 03-34-0051) were procured from Millipore (Billerica, MA, USA). DAPT (Cat. No. ab120633) was purchased from Abcam (Cambridge, UK). Other cell culture reagents and medium were from Invitrogen (Carlsbad, CA, USA).

### Primary culture of rat cortical neurons

Sprague-Dawley rats (embryonic day 16; SLC, Shizuoka, Japan) were used to prepare primary cultures of cortical neurons according to Hayashi *et al*.^34^ with minor modifications. Briefly, cerebral cortices were dissected and digested by 0.25% trypsin (Invitrogen) in phosphate-buffered saline (PBS) for 25 min at 37 °C. After trituration by use of a fire-polished Pasteur pipet in Neurobasal medium (Invitrogen) containing 10% fetal bovine serum, isolated cortical cells were suspended in Neurobasal medium containing 1 mM glutamine, 2% B27 supplement (Invitrogen), and 1% penicillin-streptomycin (Wako, Osaka, Japan). These cortical cells were plated at a density of 200,000 cells/well in 24-well plates (Falcon, Corning, NY, USA) coated with poly-D-lysine (Wako) and then cultured for 10 days before experiments. The rats were maintained according to the National Institute of Health Guide for the Care and Use of Laboratory Animals and the Guideline for Experimental Animal Care issued by the Prime Minister’s Office of Japan. All experimental procedures were approved by the Committee of Animal Care and Welfare of Tokyo University of Pharmacy and Life Sciences.

### Immunostaining

At 10 days *in vitro* (DIV), cells in primary cultures were incubated with anti-NeuN (MAB377; Millipore) and anti-GFAP (ab7260; Abcam) antibodies and then incubated with the appropriate secondary antibody, Alexa Fluor 488-labeled goat anti-mouse IgG (A11029; Invitrogen) or Alexa Fluor 594-labeled goat anti-rabbit IgG antibodies (A11037; Invitrogen). Fluorescence was detected by using an Olympus fluorescence microscope (IX-71; Olympus, Tokyo, Japan). Fluorescent images were loaded into the MetaMorph software program (Molecular Devices, Sunnyvale, CA, USA). The number of NeuN- or GFAP-positive cells was counted in 8 chosen areas of each well. Results were obtained from 2 wells in 4 independent experiments and indicated as percentages of total number of cells, which were stained with Hoechst33342 (346–07951; Dojindo, Kumamoto, Japan).

### Western Immunoblotting

For immunoblotting, cortical neurons were harvested in sample buffer comprising 62.5 mM Tris-HCl (pH 6.8), 10% glycerol, 2% SDS, and 5% β-mercaptoethanol and heated for 5 min at 95 °C. Proteins were separated by SDS-PAGE and then transferred to polyvinylidene difluoride membranes at 80 V for 1.5 h. The membranes were incubated with 5% nonfat milk in 10 mM Tris-HCl, pH 7.4, containing 0.9% NaCl and 0.1% Tween 20 for 1 h at room temperature, and then incubated overnight at 4 °C with primary antibodies. Subsequently, the membranes were probed with horseradish peroxidase-conjugated secondary antibodies (dilution, 1:5000; Pierce Biotechnology, Rockford, IL, USA). Immunoreactive proteins were detected by use of ImmunoStar basic (Wako), ImmunoStar zeta (Wako) or West Femto (Pierce Biotechnology). The following primary antibodies were used: mouse anti-β-actin (a5441, Sigma), mouse anti-GluN1 (556308, BD Biosciences, Franklin Lakes, NJ, USA), rabbit anti-GluN2A (AB1555P, Millipore), mouse anti-GluN2B (610416, BD Biosciences), and rabbit anti-calpain-2 (39165, Abcam).

### Induction of cell injury by treatment with NMDA

At 10 DIV, cortical neurons in primary culture were washed twice with 250 µl/well Hank’s balanced salt solution (HBSS; Invitrogen) containing 2.4 mM CaCl_2_ and 20 mM HEPES without magnesium, which can block the NMDA receptor (HBSS buffer). The neurons were incubated for 15 min at 37 °C between each wash. Subsequently, the neurons were incubated with the desired concentration of NMDA and 10 µM glycine, a co-activator of the NMDA receptor, in HBSS containing 2.4 mM CaCl_2_ and 20 mM HEPES without magnesium for 15 min at 37 °C. After treatment with or without NMDA, cortical neurons were cultured for the desired times in the culture medium. As the control experiments for NMDA treatment, cortical neurons were incubated with HBSS buffer lacking both NMDA and glycine. Inhibitors for furin, γ-secretase (DAPT), matrix metalloproteinase (GM6001), and PCSK9 (SBC115076) were added at the desired concentration 24 h before the addition of NMDA. NMDA receptor antagonist MK-801 was incubated for 15 min with NMDA at the same time. Calpeptin, which is a potent calpain inhibitor, was added 6 h before the addition of NMDA. In the results, age-matched cultured cortical cells without any treatment were used as the “untreated control group.”

### Measurement of intracellular Ca^2+^

The cortical neurons were first incubated with 3 µM Fluo-8 acetoxymethyl ester (AAT Bioquest, Sunnyvale, CA, USA) for 30 min at 37 °C and then washed twice with HBSS containing 2.4 mM CaCl_2_, 20 mM HEPES without magnesium, after which 30 µM NMDA and 10 µM glycine were added. Continuous fluorescent images were taken every 500 ms by an ORCA-R2 digital CCD camera (Hamamatsu Photonics, Hamamatsu, Japan) attached to an Olympus IX71 microscope (Olympus) and analyzed by using MetaFluor fluorescence ratio imaging software (Molecular Devices).

### Cell viability assay

Cell viability of the cortical neurons was determined by the XTT dye-reduction assay as previously described^35^ with minor modifications. The neurons were incubated with 250 µg/ml XTT and 6.25 µM 1-methoxy-5-methylphenazinium methyl sulfate in culture medium for 1 h at 37 °C. Then, the culture media were transferred to a 96-well assay plate (Corning) for measurement. The absorbance at 450 nm was measured with a plate reader (EMax Plus Microplate Reader, Molecular Devices). The relative cell viability was expressed as the ratio of the absorbance at 450 nm of each treatment group against that of the corresponding untreated control group.

### Calpain-Glo^TM^ protease assay

Calpain activity in the cortical neurons was measured by performing the Calpain-Glo^TM^ protease assay (Promega, Fitchburg, Wisconsin, USA) according to the manufacturer’s instructions. Briefly, a volume of Calpain-Glo reagent equal to that of the medium in cortical neuron cultures was added. Then, the neurons were incubated for 10 min at 37 °C. Culture supernatants were transferred to a white 96-well plate for measurement, and luminescence was measured with a plate-reading luminometer, ARVO X2 (PerkinElmer, Waltham, MA, USA).

### Statistical analysis

All data were presented as the means ± standard error of the mean. Statistical analyses among multiple groups were performed by using analysis of variance followed by the Tukey test as a *post hoc* test. P values of less than 0.05 were considered to indicate statistical significance.

## Electronic supplementary material

Supplementary Information
