# Supplementary material for: Furin inhibitor protects against neuronal cell death induced by activated NMDA receptors
Source: Sci Rep. 2018 Mar 26;8:5212. doi: 10.1038/s41598-018-23567-0 (PMC5980093; doi:10.1038/s41598-018-23567-0)
Supplement: Supplementary file 1 — Supplementary Information [file 41598_2018_23567_MOESM1_ESM.pdf]

Supplementary Information

Furin inhibitor protects against neuronal cell death induced by activated NMDA receptors

Mariko Yamada, Hideki Hayashi, Moe Yuuki, Nahoko Matsushima, Bo Yuan, and Norio Takagi

Department of Applied Biochemistry, Tokyo University of Pharmacy and Life Sciences, 1432-1 Horinouchi, Hachioji, Tokyo 192-0392, Japan.

Address correspondence and reprint requests to Dr. Norio Takagi, Department of Applied Biochemistry, Tokyo University of Pharmacy and Life Sciences, 1432-1 Horinouchi, Hachioji, Tokyo 192-0392, Japan. e-mail: takagino@toyaku.ac.jp

Supplemental materials and methods

The direct effect of calpeptin and furin inhibitor on calpain activity.

To examine direct effect of furin inhibitor on calpain activity *in vitro*, calpain-1 (Cat. No. 208713) were procured from Millipore (Billerica, MA, USA). The effects of calpeptin (calpain; Ki = 7 nM) and furin inhibitor 1 (FI-1 : furin; Ki = 1 nM) on activity of recombinant calpain were measured by performing the Calpain-Glo^{TM} protease assay according to the manufacturer's instructions. Briefly, a volume of Calpain-Glo reagent equal to that of the indicated concentration of calpeptin or furin inhibitor in recombinant calpain (10 nM) was added. Then, they were incubated for 10 min at 37 ºC. Luminescence was measured with a plate-reading luminometer, ARVO X2 (PerkinElmer, Waltham, MA, USA). The doses (10, 30, and 100 nM) used in this experiment were based on the Ki values for calpeptin and furin inhibitor 1 (FI-1).

S1

Figure 1 (c)

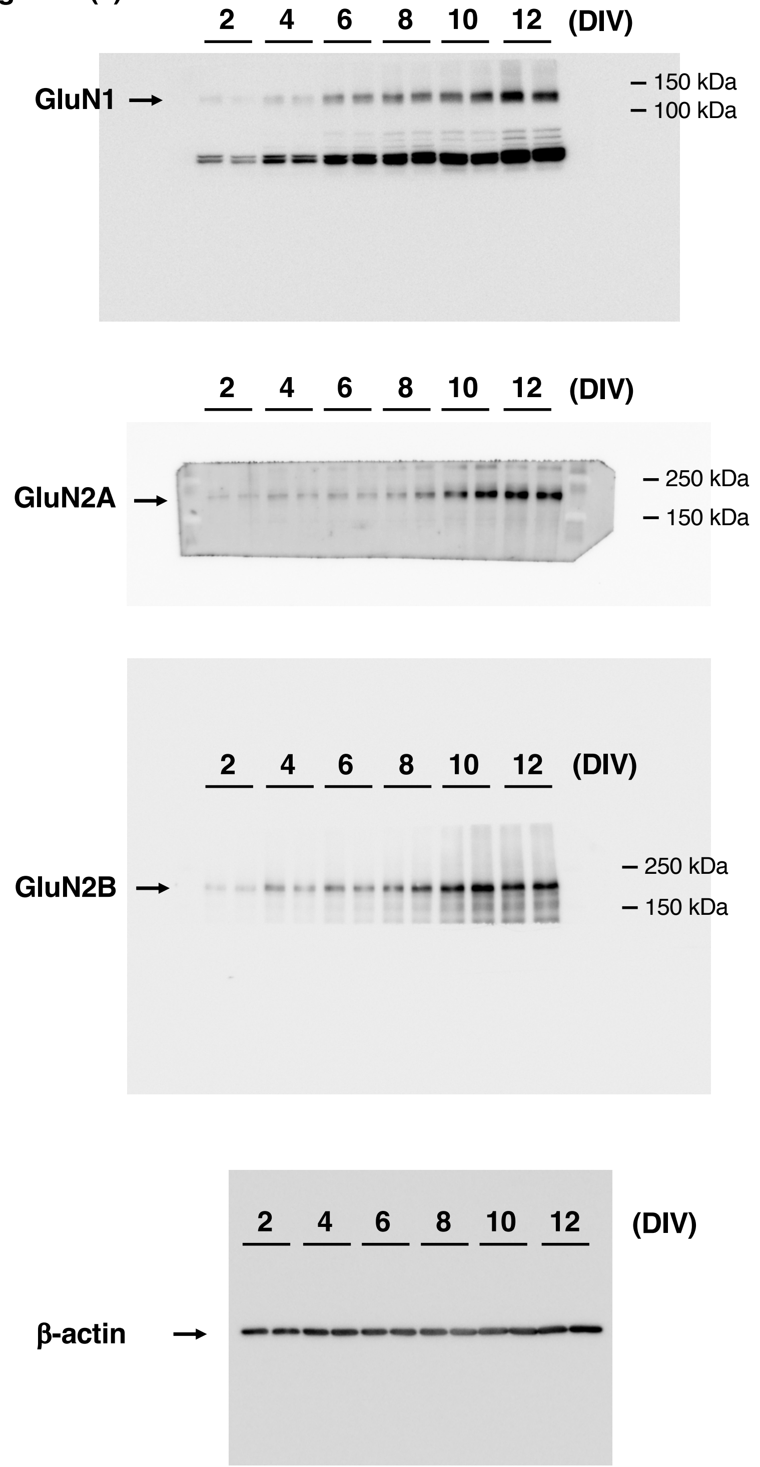

Supplementary Figure S1. Full-length blots corresponding to crop blots presented in the main manuscript

Full-length blots for Figure 1C (GluN1, GluN2A, GluN2B and actin). Area displayed in the main manuscript delineated by red rectangle.

S2

Figure 5 (a)

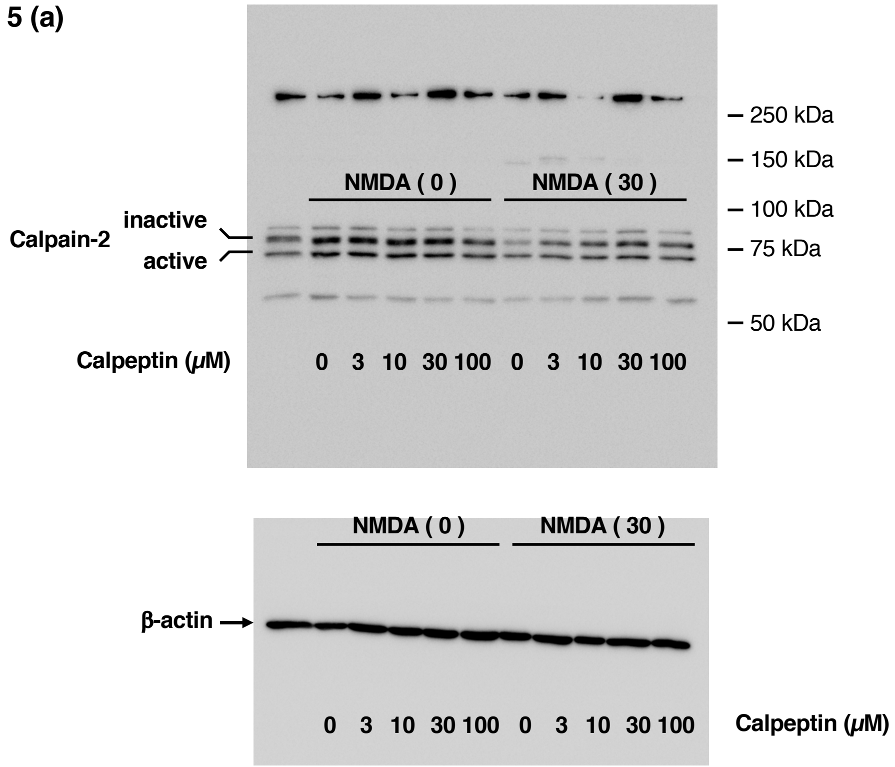

Figure 5 (b)

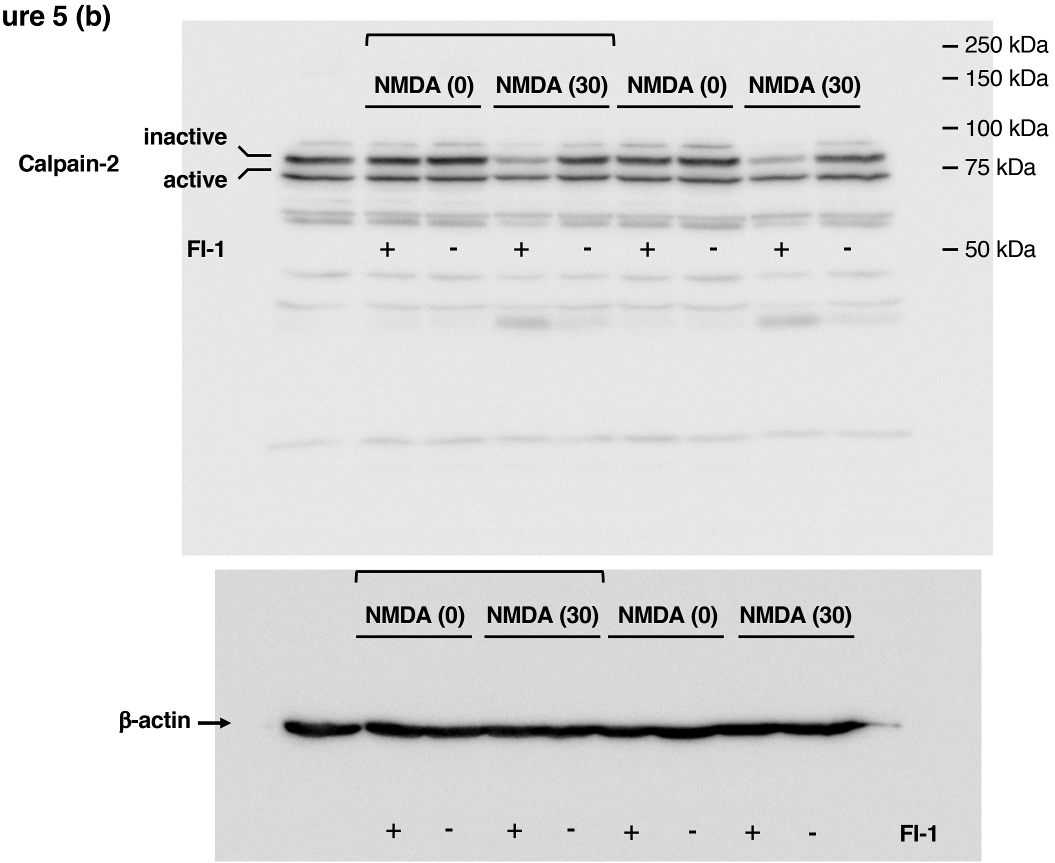

Supplementary Figure S2. Full-length blots corresponding to crop blots presented in the main manuscript.

Full-length blots for Figure 5A and 5B. Area displayed in the main manuscript delineated by red rectangle.

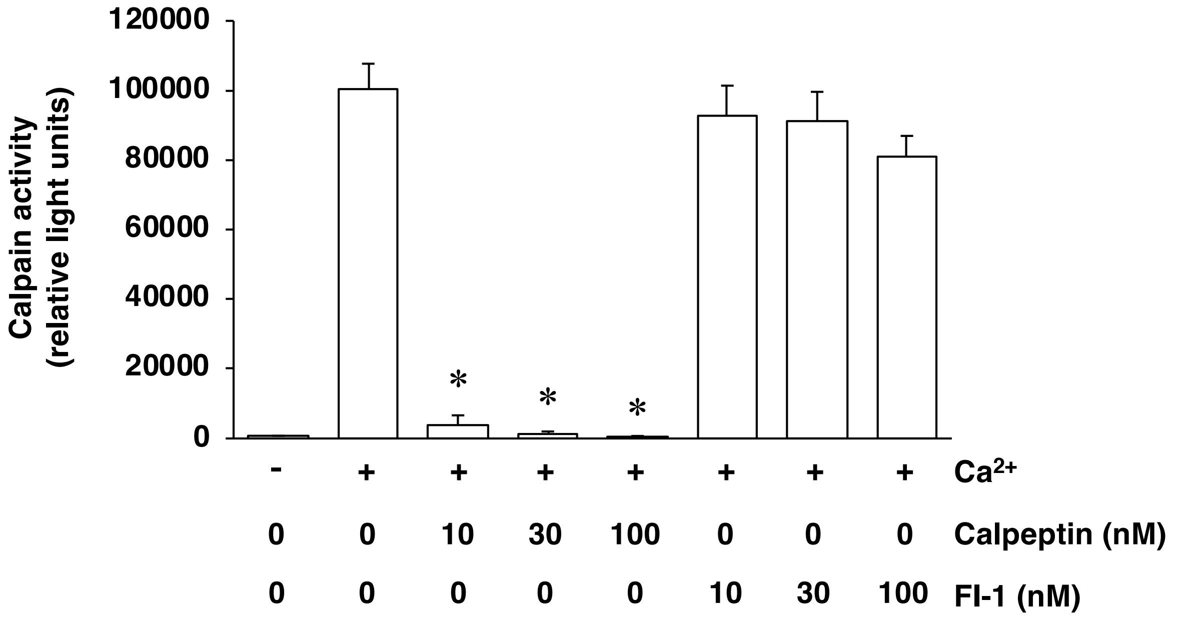

Supplementary Figure S3. The direct effect of calpeptin and FI-1 on the calpain activity. Direct effect of calpeptin and furin inhibitor 1 (FI1) on calpain activity in the presence of Ca^{2+}. The activity of recombinant calpain treated with 0, 10, 30 and 100 nM calpeptin or furin inhibitor 1 is shown. Calpain activity was measured by performing the Calpain-Glo™ protease assay and expressed as the percentage of the inhibitor-untreated control group. Results are the means ± SE (n=3 independent experiments). * indicates a significant difference from the inhibitor-untreated group ($p < 0.05$).
